# Highly Selective Copper Ion Imprinted Clay/Polymer Nanocomposites Prepared by Visible Light Initiated Radical Photopolymerization

**DOI:** 10.3390/polym11020286

**Published:** 2019-02-08

**Authors:** Radhia Msaadi, Gorkem Yilmaz, Andrit Allushi, Sena Hamadi, Salah Ammar, Mohamed M. Chehimi, Yusuf Yagci

**Affiliations:** 1 Faculté des Sciences, Unité de Recherche Électrochimie, Matériaux et Environnement UREME (UR17ES45), Université de Gabès, 6000 Gabès, Tunisia; radhiaradhia44@gmail.com (R.M.); salah.ammar@fsg.rnu.tn (S.A.); 2 Department of Chemistry, Maslak, IstanbulTechnical University, 34469 Istanbul, Turkey; a.gorkemyilmaz@gmail.com (G.Y.); andrit.allushi@chem.lu.se (A.A.); 3 ICMPE (UMR 7182), CNRS, UPEC, Université Paris Est, F-94320 Thiais, France; hamadi@icmpe.cnrs.fr

**Keywords:** clay, diazonium salt, ion imprinted polymers, radical photopolymerization, visible light, adsorption, copper ions

## Abstract

There is an urgent demand worldwide for the development of highly selective adsorbents and sensors of heavy metal ions and other organic pollutants. Within these environmental and public health frameworks, we are combining the salient features of clays and chelatant polymers to design selective metal ion adsorbents. Towards this end, the ion imprinting approach has been used to develop a novel nanohybrid material for the selective separation of Cu^2+^ ions in an aqueous solution. The Cu^2+^-imprinted polymer/montmorillonite (IIP/Mt) and non-imprinted polymer/montmorillonite (NIP/Mt) nanocomposites were prepared by a radical photopolymerization process in visible light. The ion imprinting step was indeed important as the recognition of copper ions by IIP/Mt was significantly superior to that of NIP/Mt, i.e., the reference nanocomposite synthesized in the same way but in the absence of Cu^2+^ ions. The adsorption process as batch study was investigated under the experimental condition affecting same parameters such as contact time, concentration of metal ions, and pH. The adsorption capacity of Cu^2+^ ions is maximized at pH 5. Removal of Cu^2+^ ion achieved equilibrium within 15 min; the results obtained were found to be fitted by the pseudo-second-order kinetics model. The equilibrium process was well described by the Langmuir isothermal model and the maximum adsorption capacity was found to be 23.6 mg/g. This is the first report on the design of imprinted polymer nanocomposites using Type II radical initiators under visible light in the presence of clay intercalated with hydrogen donor diazonium. The method is original, simple and efficient; it opens up new horizons in the general domain of clay/polymer nanocomposites.

## 1. Introduction 

There is global concern about heavy metal pollution of the environment considering the high risk of these elements entering the food chain via natural and anthropogenic routes [1]. Heavy metal ion pollutants affect humans after ingestion of water and food [2,3]. For example, children are particularly vulnerable to the neurotoxic effects of lead, and exposure, even at relatively low levels, may cause serious, and in some cases irreversible, neurological damage [4].

For these reasons, heavy metal pollution is regarded as one of the most challenging global problems and requires combined actions such as detection at minute levels and removal at a separation/filtration stage using high-capacity selective adsorbents. The first issue can be addressed by developing highly sensitive and selective sensors for a YES/NO response of the sensors to indicate whether or not a sample is contaminated by heavy metals; ideally the output response would be processed to return a quantitative value of the metal ion concentration in the target sample [5,6]. The second important issue is to develop high-capacity adsorbents to remove or pre-concentrate heavy metals in order to have a complete picture of the pollution by metal ions from the surface physicochemical point of view. Towards this end, clays can be regarded as natural inorganic “sponges” to retain heavy metal ions [7,8]. However, aluminosilicates are negatively charged and retention of heavy metal ions could be achieved through cation exchange reactions. In this sense, clays are not selective [8] and organo-modification impart to clays functional groups for the specific retention of metal ions. For example, NH_2_, SH, C=C, calixarene, crown-ethers and cryptand, dendrimer among others have been attached to clays via ammonium salts, diazonium salts, silane coupling agents, or other strategies [9,10,11,12,13,14,15,16,17]. In a more elaborate picture, clay/polymer nanocomposites can also be envisaged if the polymer possesses pendant functional groups able to complex metal ions [18], yet selectivity remains challenging. Some studies have reported various synthetic approaches for the synthesis of clay-polymer nanocomposites including free radical, cationic and ring-opening polymerizations, and click chemistry [19,20,21,22,23]. By incorporating suitable functional groups onto the clay surface, a wide variety of polymerization systems can successfully be applied in an in situ manner.

In order to further push the frontiers of selectivity, a smart strategy consists of making clay/ion imprinted polymer nanocomposites [24,25], therefore combining the best of both worlds: high-capacity mineral adsorbent [26] and highly selective organic material *c.a*. ion-imprinted polymers [27,28,29,30,31]. In brief, molecularly imprinted polymers (MIPs) are prepared by in situ co-polymerization of vinylic monomers and crosslinkers or using conjugated monomers in the presence of template molecules. Crosslinking traps the template molecules in the polymeric network. Upon washing with appropriate strong solvents, template molecules are removed and the polymeric network is left with molecular imprints that serve as artificial receptor sites for selectively rebinding the templates. The same strategy can be envisaged for the making of ion imprinted polymers (IIPs), i.e., for the synthesis of a crosslinked network in the presence of metal ions or metal ion complexes. There is a wealth of strategies reported in the literature on the preparation of MIPs and IIPs, as well as their nanocomposites, which are described in [25,27,32,33,34,35]. We have previously demonstrated that such materials can be designed by UV-triggered radical photopolymerization of functional and crosslinker in the presence of organoclay [36]. In a unique approach, we have efficiently organo-modified clay using the versatile aryl diazonium instead of ammonium salts or silane coupling agents bearing photoinitiator.

Yet, the original design of nanohybrids we have reported recently requires UV light initiation. Instead, herein we simplify the process by triggering radical photopolymerization under visible light using diazonium-modified clay as a macro-initiator and camphorquinone (CQ) as a photosensitizer. CQ is indeed a very efficient photosensitizer for generating ultrathin polymer coatings by simulated sunlight-induced surface-confined radical photopolymerization [37]. To the best of our knowledge, this strategy has never been reported previously; it is highly interesting simply because the clay/IIP nanocomposite can be designed using natural or simulated daylight or white light.

Herein, montmorillonite was first cation exchanged with a diazonium compound bearing dimethyl amino groups to provide a macro-photoinitiator, and visible light radical photopolymerization was conducted in the presence of the modified clay, the monomers (4-vinylpyridine, (VP) methacrylic acid (MA), CQ, and the heavy metal ions. Thorough rinsing of the final product removed the metal ions and created artificial receptor sites for the selective recognition of copper.

## 2. Experimental

### 2.1. Chemicals and Reagents 

All chemical reagents and materials were purchased from Aldrich (Steinheim, Germany) and used without further purification. Milli-Q Plus water purification system (Millipore, Billerica, MA, USA) was employed to prepare deionized water.

### 2.2. Instrumentation and Characterization 

X’Pert PRO (PANalytical, Almelo, The Netherlands) instrument fitted with a Co Kα X-ray source (1.789 Ǻ) was employed to record the diffraction patterns of the samples.

Infrared spectra were recorded using a Nicolet Magna-IR 550 spectrometer (Thermo Nicolet, Madison, WI, USA) in the attenuated total reflection (ATR) mode, in the 4000–450 cm^−1^ range.

AK Alpha instrument (Thermo, East-Grinstead, UK) fitted with a monochromated Al Kα X-ray source (*hν* = 1486.6 eV, spot size = 400 µm) was used for XPS measurements. A flood gun was employed for static charge compensation. The analyzer was operated at 80 and 200 eV pass energy for the narrow regions and survey spectra, respectively. Elemental atomic concentrations were computed using the integrated peak areas and the corresponding sensitivity factors provided by the manufacturer.

The determination of metal ion concentration was performed with Avanta/GBC flame atomic absorption spectrophotometer (Reutlingen, Germany) equipped with a hollow cathode lamp and deuterium background corrector at respective wavelength resonance line using an air-acetylene flame.

### 2.3. Synthesis of the Diazonium Salt Cl^−^ N_2_ C_6_H_4_N=NC_6_H_4_N-(CH_3_)_2_

*N,N*-Dimethy-4,4’azoanilinediazonium salt was prepared as follows: the aromatic amine (4.16 mmol, 1 g) is suspended in a hydrochloric acid and distilled water 50/50 *v*/*v*%. The mixture was cooled in an ice bath, and then 1 equivalent of sodium nitrite was slowly added. The mixture was stirred for 15 min. IR characterization of the diazonium salt $N_{2}^{+}$, 2204 cm^−1^.

### 2.4. Diazonium Modification of Montmorillonite

To an aqueous suspension of clay (100 mg dispersed in 10 mL of de-ionized water) we added dropwise an aqueous solution of diazonium salt (500 mg in 10 mL of deionized water). The intercalated clay was washed thoroughly with deionized water and dried at 60 °C overnight.

### 2.5. Synthesis of Cu^2+^_IIP/Mt Nanocomposite

The IIP/Mt nanocomposite was prepared in two major sequential steps. First, 1 equivalent of template Cu(NO_3_)_2_, 2 equivalents of 4-vinylpyridine (4-VP, 0.431 mL) and 2 equivalents of methacrylic acid (MA, 0.339 mL)were dissolved in (4 mL/4 mL *v*/*v*) DMF and water in a glass tube, and were kept under stirring for 1 h. Then, 1 equivalent of crosslinker ethylene glycol dimethacrylate (EDGMA, *V* = 0.377 mL), 41 mg of CQ (8 wt % of the monomer 4-VP or methacrylic acid MAA) and 200 mg of modified clay (Mt-DZ) by diazonium (dimethyl-4,4-azoaniline diazonium) were added in glass tube. The glass tube was purged with nitrogen for 15 min. The mixture was irradiated using a Ker-Vis Blue photoreactor (Istanbul, Turkey) equipped with six lamps (Philips Tl-D 18 W, supplied with photoreactors) emitting light nominally at 400–500 nm at room temperature for 4 h.

Template ion (Cu^2+^) was removed by washing the nanocomposite in HNO_3_ (0.1 M) in order to obtain specific artificial recognition sites within the IIP/Mt nanocomposites.

The same clay-polymer nanocomposite was prepared but in the absence of copper; it is noted NIP/Mt for non-imprinted polymer/montmorillonite.

### 2.6. Metal Ion Adsorption

Adsorption of Cu^2+^ on the IIP/Mt nanocomposite and NIP/Mt nanocomposite was examined using batch experiments. To prepare adsorption isotherms, a series of samples containing 4 mg of an appropriate IIP/Mt and NIP/Mt as equilibrated with 10 mL solution containing various concentrations (5–20 mg/L) of Cu^2+^. The solution was shaken at room temperature for 2 h. After adsorption, the mixture was isolated by centrifugation, then filtered through a 0.45 µm membrane filter and examined with atomic absorption spectroscopy (AAS). The adsorption amount (*q_e_*; mg/g) was calculated using:

(1) $$q_{e}=\frac{(C_{0}-C_{e})V}{m},$$

where $C_{0}$ and $C_{e}$. are initial and equilibrium concentration (mg/L), *m* is the sorbent mass (g), and *V* is the solution volume (L).

Kinetic studies were conducted to determine the adsorption rate of Cu^2+^ from water samples as follows: 4 mg of each sorbent were dispersed in 10 mL solution containing the same initial Cu^2+^ concentration of 20 mg/L. Each mixture was continuously batch oscillated at room temperature for 5, 10, 15, 30, 60, 90, and 120 min. After each time period, solutions were filtered and analyzed by AAS to determine the concentration of Cu^2+^ in the supernatant.

To investigate the effect of pH, 4 mg IIP/Mt and NIP/Mt were added to 20 mL sample solutions containing 20 mg/L of Cu(II) ion at pH range 2.0–9.0, respectively, pH values were adjusted by 1 mol/L HNO_3_ and 1 mol/L NaOH.

### 2.7. Selectivity Experiments

To estimate the selectivity of the ion-imprinted polymer/Mt nanocomposite and their corresponding non-imprinted polymer/Mt nanocomposites materials for copper adsorption, 5 mg of the sorbent was added into 10 mL of 20 mg/L binary solution of Cu^2+^/Zn^2+^, Cu^2+^/Pb^2+^, and Cu^2+^/Fe^3+^ for 1 h. The mixture was magnetically stirred at 300 rpm, then left to decant for 15 min, centrifuged, and filtered. Finally, the concentration of copper and the interfering ions was measured by AAS.

The distribution coefficients, *K_d_* (mL/g), of Cu^2+^, Zn^2+^, Pb^2+^, and Fe^3+^ were calculated using the equation:

(2) $$K_{d}=\frac{\left(C_{0}-C_{e}\right)\;V}{C_{e}\;m}.$$

The selectivity coefficients for the binding of a copper ion in the presence of other competing ions in binary systems were calculated according to the following equations:

(3) $$K=\frac{K_{d}\left(Cu^{2+}\right)}{K_{d}\left(M^{n+}\right)},$$

where *M^n+^* (*n* = 2 or 3) represents Cu^2+^ competing ions mentioned above. The coefficients give an indication as to how selective the nanocomposite is for Cu^2+^ ions in the presence of other competing species in solution.

The relative selective coefficient *K′*, which represents the enhanced effect of imprinting on selectivity and adsorption affinity for the template onto the ion imprinted polymer/montmorillonite nanocomposite. The *K′* of the IIP/Mt nanocomposite against the NIP/Mt nanocomposite as determined using the equation:

(4) $$K^{\prime }=\frac{K_{\mathrm{IIP}/\mathrm{Mt}}}{K_{\mathrm{NIP}/\mathrm{Mt}}},$$

where *K*_IIP/Mt_ and *K*_NIP/Mt_ are the selectivity coefficients of the IIP/Mt and NIP/Mt, respectively.

### 2.8. Desorption and Regeneration

To estimate the reuse ability of IIP/Mt nanocomposite, synthesized adsorbent were contacted with Cu^2+^ solution for adsorption process. After adsorption, the IIP/Mt were placed in the desorption medium (1 M, HNO_3_ solution) and stirred for 120 min at room temperature. This procedure was repeated for many times until Cu^2+^ could not be detected in aqueous phase. Then, the adsorbent was washed thoroughly with double distilled water to a neutral pH to determine reusability of IIP/Mt nanocomposite, the desorption-adsorption procedure was repeated eight times using the same sorbent.

## 3. Results and Discussion

### 3.1. Synthesis Strategy and Mechanistic Aspects

**Scheme 1:**
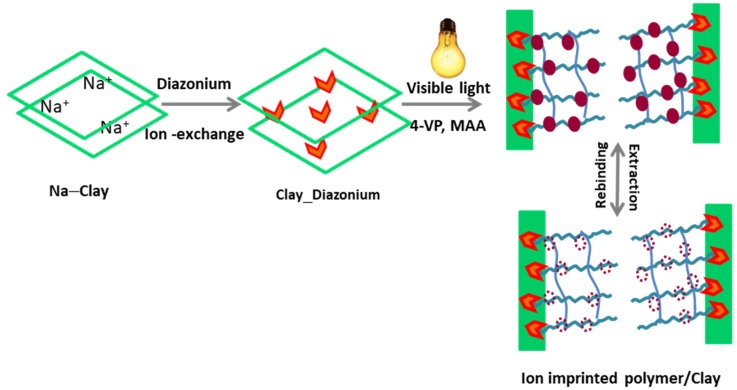
Schematic representation of the synthesis procedure of Cu^2+^_IIP/clay nanocomposite. NB: Under irradiation conditions the azo compound undergoes cis-trans isomerization. In fact, this behavior would be more beneficial since it would lead to a better intercalation.

The synthesis strategy of IIP/Mt nanocomposites was carried out in two main steps as illustrated in Scheme 1, starting by a simple diazonium cation exchange reaction with Na^+^. Actually, when Na–clay is dipped in a diazonium aqueous solution, the diazonium cation replaces Na^+^ instantaneously [11,12]. Sodium is depleted and clay swells due to the intercalation of the diazonium; such a swelling is visible with the naked eye. The cation exchange reaction usually leads to aluminosilicate-diazonium complex or diazoether of the type Si–O–N=N–aryl, which, upon heating at 60 °C, undergoes dediazotization and yields stable aluminosilicate-aryl bond of the type Si–O–aryl [36].

**Scheme 2:**
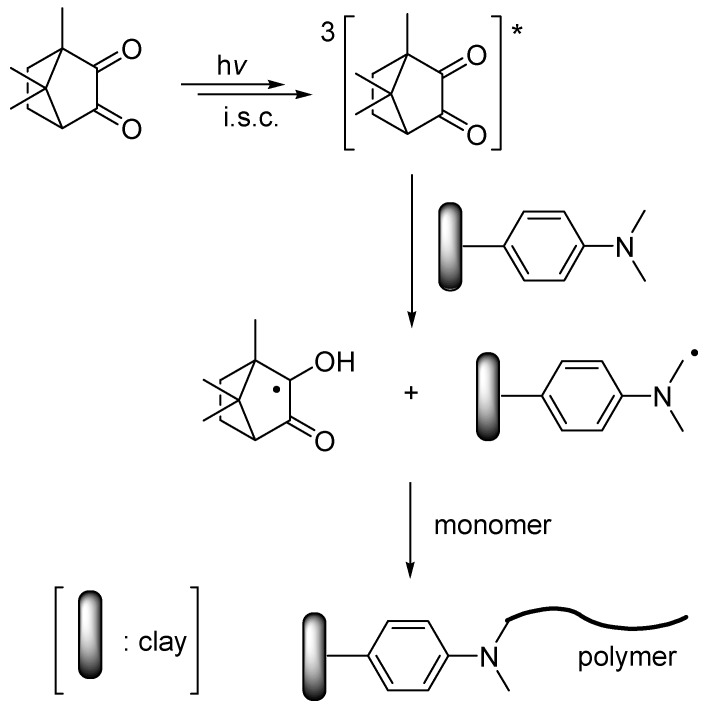
Mechanism of visible light photopolymerization on the clay surface by using camhorquinone. The structure of the attached azoic initiator is simplified for the sake of clarity.

For the photopolymerization step, we have deliberately selected CQ as the photosensitizer for two reasons. First, it absorbs the light in the visible region of the electromagnetic radiation. Although there are many visible light acting photoinitiating systems [38,39,40], CQ is the safest photoinitiator and therefore, widely used in dental applications. Secondly, upon photolysis, the triplet CQ abstracts hydrogen from hydrogen donors, i.e., amines. The amine-derived radicals that initiate the polymerization CQ–H^·^ radicals are not reactive towards monomers, but readily form various coupling products. This is particularly important for our case as chain growth occurs only on the clay surface. The overall mechanism is presented in Scheme 2.

Separately, to form the pre-polymerization complex, Cu(NO_3_)_2_ (1 equivalent) as mixed with 4-vinylpyridine (1 equiv), acrylic acid (2 equiv) in DMF/H_2_O (4 mL/4 mL) and the mixture was shaken for 1 h. This solution was then mixed with EGDMA (1 equiv), diazonium-modified clay Mt_DZ (100 mg) and camphorquinone (26.6 mg). The suspension was purged with nitrogen for 15 min in order to prevent any unwanted reactions that inhibit the photopolymerization process. The mixture was irradiated using a Ker-Vis blue photoreactor equipped with six lamps (Philips TL-D 18 W) emitting light nominally at 400–500 nm at room temperature for 4 h.

### 3.2. Characterization

#### 3.2.1. XRD

**Figure 1:**
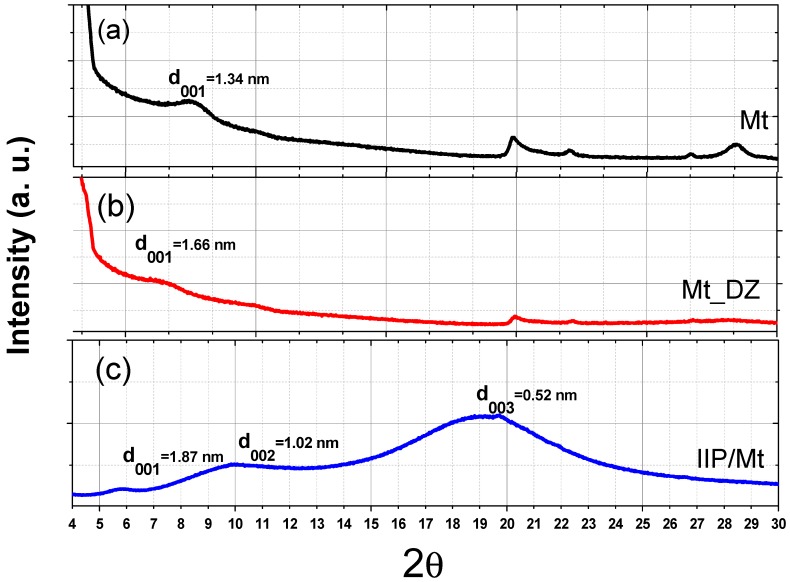
XRD patterns of montmorillonite Mt (**a**), Mt_DZ (**b**) and IIP/Mt (**c**) nanocomposite.

X-ray powder diffraction diagrams (DRX) for sodium montmorillonite clay (Mt), montmorillonite interposed with diazonium salt (Mt_DZ) and montmorillonite after photopolymerization (IIP/Mt) are shown in Figure 1.

For (Mt_DZ), the *d*_001_ peak position is shifted to lower values (Figure 1b) compared to the untreated clay (Figure 1a). The *d*-spacing of soda montmorillonite (Mt), modified montmorillonite (Mt_DZ) and montmorillonite/ionic imprinted polymer nanocomposite (IIP/Mt) were estimated from the peak (001) using the Bragg equation *n*λ = 2*d*, sinθ. After the cation exchange reaction between the Na^+^ cations and the diazonium salt, the basal distance was increased from 1.34 nm to 1.66 nm, which proves the intercalation of diazonium salt between the montmorillonite layers. After photopolymerization the distance d_001_ moves to the lower value of 2θ (*d*_001_ = 1.8 nm), which means that after the in-situ radical photopolymerization the polymer chain is intercalated between the clay layers [41]. It is to note that Figure 1c showing the X-ray diffraction of the IIP/Mt nanocomposite, suggests that this adsorbent is characterized by (00l) (1 < *l* < 4) reflection, which testifies to the regular distribution of the polymer within the clay nanosheets. The positions (001), (002) and (003) peaks at *d* = 1.87, 1.02 and 0.52 nm, respectively, indicate an intercalated crystalline structure of the IIP in the interlayers.

#### 3.2.2. FTIR Analysis

**Figure 2:**
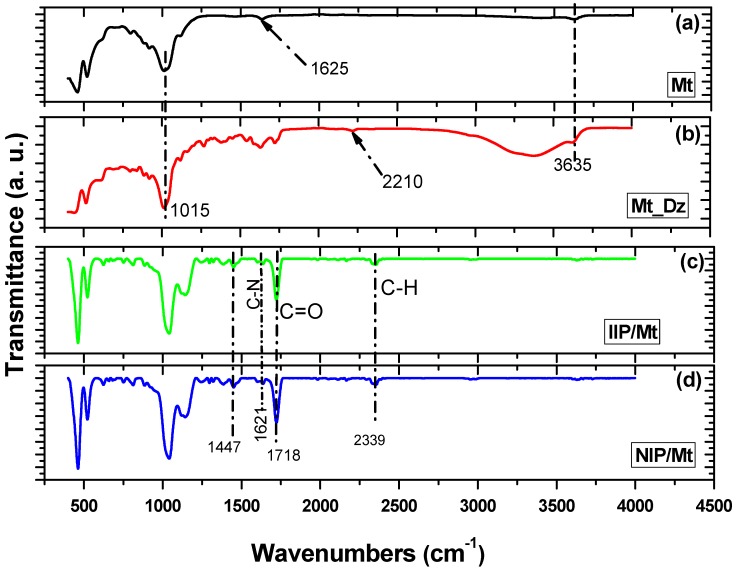
FTIR spectra of Mt (**a**), diazonium-modified montmorillonite, Mt_DZ (**b**), IIP/Mt (**c**), and NIP/Mt (**d**).

Figure 2 shows the FTIR spectra of Mt, Mt_DZ, IIP/Mt and NIP/Mt. The structure of the purified montmorillonite is characterized by the bands located in the 1000–1200 cm^−1^ range assigned to Si–O–Si (Figure 2a). The peaks at 3635 and 918 cm^−1^ are in line with the dominant presence of dioctahedral smectite with (Al, Al–OH) [42]. The absorption band at 1625 cm^−1^ corresponds to OH from the water molecules absorbed by the clay. After clay modification the FTIR analysis confirmed the presence of diazonium in the clay inter-spacing (Figure 2b). FTIR data of modified clay with diazonium prove $N_{2}^{+}$ band at 2210 cm^−1^. Moreover, after clay modification we note the appearance of a band at 1621 cm^−1^ assigned to the pyridine ring, and an intense band at 1447 cm^−1^ ascribed to the stretching vibration of C–N of 4-vinylpyidine. One can also note the presence of a new signal near 1718 cm^−1^ corresponding to the carbonyl of the methacrylic acid (Figure 2c). These FTIR observations are in line with the coexistence of an organic polymer fraction in the final composite material. Figure 2d displays the IR spectrum of NIP/Mt; it is similar to that of the IIP/Mt. It follows that one can confidently compare the capture of the template ion by IIP/Mt and NIP/Mt as they have the same chemical structure. The copper removal results could thus be related to the existence of copper artificial receptors within IIP/Mt, or their absence in the case of NIP/Mt.

#### 3.2.3. XPS Analysis

**Figure 3:**
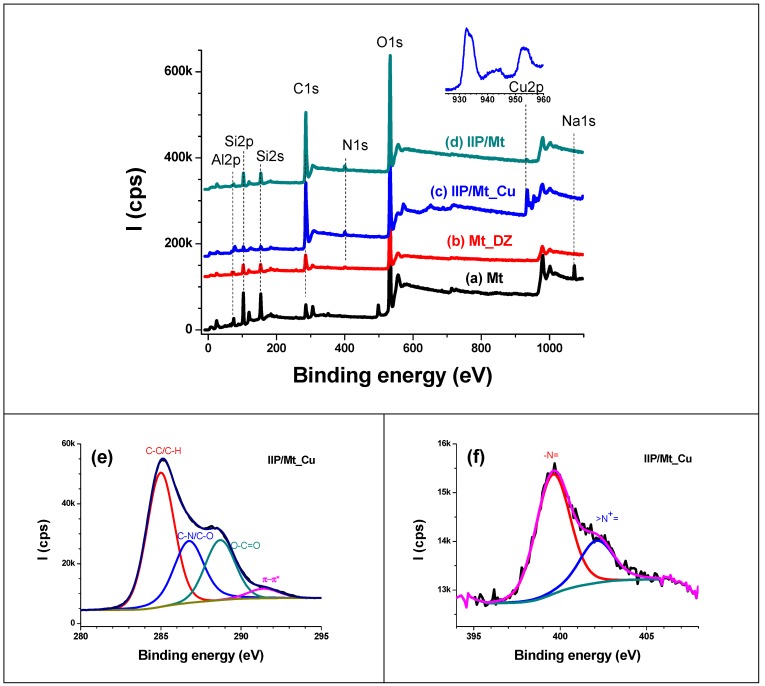
XPS analysis of Mt, Mt_DZ and IIP/Mt_Cu (before extraction of copper) and IIP/Mt (after extraction of copper). Survey regions: (**a**–**d**), peak-fitted C1s and N1s narrow regions of IIP/Mt_Cu: (**e**,**f**), respectively.

Figure 3 displays XP spectra of Mt, Mt_DZ, and the nanocomposites before and after extraction of copper. The survey regions are displayed in Figure 3a–d; and the main peaks are Al2p (~74 eV), Si2p (~102 eV), Si2s (152 eV), C1s (285 eV), N1s (400 eV), O1s (532 eV), Cu2p (930–960 eV) and Na1s (1072 eV). It is demonstrated here that the diazonium salt intercalates montmorillonite by cation exchange mechanism since one can see in Figure 3b the disappearance of the Na1s peak compared to the pristine clay (Figure 3a). The intercalation leads also to an increase in the C1s/Si2p intensity ratio and appearance of a tiny N1s peak (~400 eV) from the intercalated diazonium. The synthesis of the IIP/Mt_Cu results in a significance change in the survey region (Figure 3c) which exhibits sharp C1s and O1s peaks from the polymer and the existence of a Cu2p doublet. The high resolution Cu2p from IIP/Mt_Cu is shown in insert. Nitrogen is also slightly more visible as vinylpyridine was used here as a comonomer. After extraction, the Cu2p vanishes (Figure 3d). Although the results discussed below indicate significant adsorptive behavior of IIP/Mt compared to NIP/Mt, it would be interesting to compare the extents of copper after adsorption on the imprinted and the non-imprinted supports.

Figure 3e,f display the high resolution C1s and N1s peaks of the IIP. Interestingly, the C1s shows features due to C–N (~286 eV) and the functional group COOH (peak component at 289 eV). As far as the peak component at 292 eV is concerned, it is ascribed to aromatic species from the vinylpyridine repeat units and the diazonium-derived aryl groups attached to the clay sheets. The N1s feature from the IIP (Figure 3f) shows free and quaternized nitrogen species. The latter could be due to protonation by the COOH group of the methacrylic acid or by the OH groups from clay.

### 3.3. Adsorption of Cu^2+^

#### 3.3.1. Effect of pH

**Figure 4:**
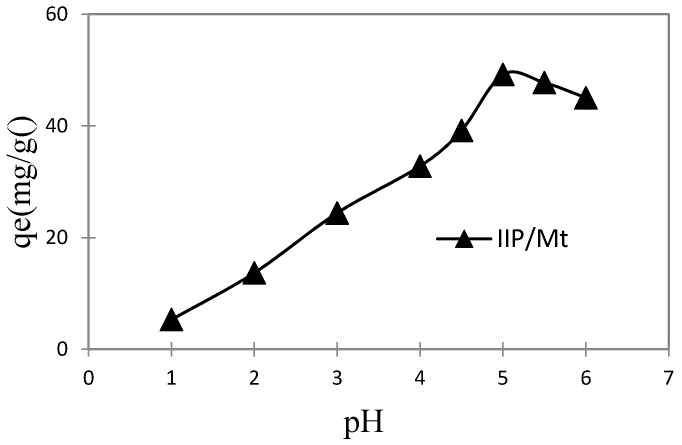
Effect of pH on adsorption capacity of IIP/Mt. Cu^2+^ initial concentration: 20 mg/L; adsorption time: 2 h; IIP/Mt: 4 mg; room temperature.

The pH of the solution has a significant influence on the adsorption process as it is well-known to affect the protonation of surface functional groups. Figure 4 displays the adsorption capacity of Cu^2+^ by the IIP/Mt support; the maximum is noted at pH 5. Above this value, adsorption starts to decrease due to precipitation of copper [43]. For low pH values the adsorption of copper ions is also low this could be due to the protonation of functional sites and consequently a competition between protons from the acidic medium and Cu^2+^ ions towards the artificial receptor sites of copper. In acidic medium, adsorption increases with pH as reported for clays [44,45].

#### 3.3.2. Effect of Contact Time and a Kinetic Study

**Figure 5:**
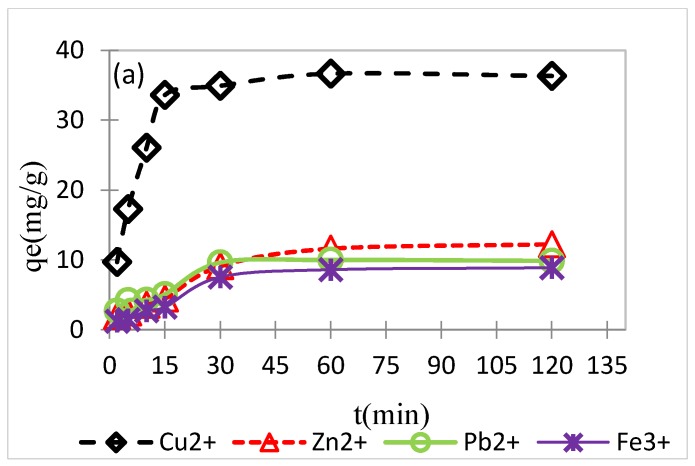

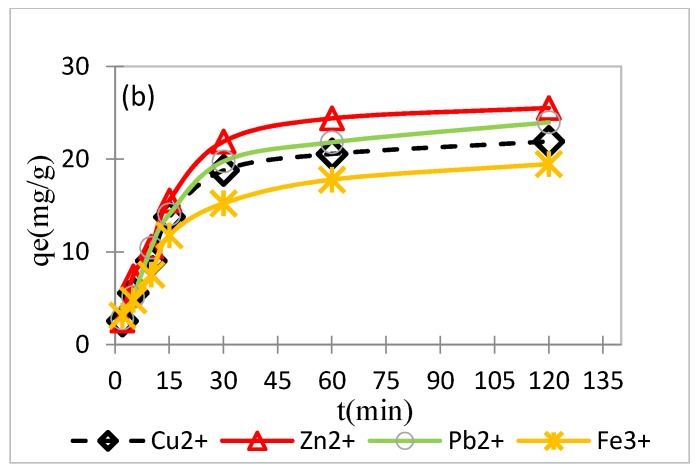
Effect of contact time on the removal of Cu^2+^, Zn^2+^, Pb^2+^, and Fe^3+^ by IIP/Mt (**a**), and NIP/Mt (**b**). Conditions: initial Cu^2+^ concentration = 20 mg/L, and pH = 5.

The effect of contact time on the adsorption of Cu^2+^, Zn^2+^, Pb^2+^ and Fe^3+^ on IIP/Mt and NIP/Mt nanocomposites is displayed in Figure 5. The adsorption of Cu^2+^ ions is rapid; the adsorption capacity increases with time and reaches a maximum during the first 15 min, then increases to saturation. The extent of adsorption of Cu^2+^ is much higher than that of Zn^2+^, Pb^2+^ and Fe^3+^ ions (Figure 5a). This rapid equilibrium could be explained by the high affinity of complexation and geometry between Cu^2+^ ions and the cavities of the nanocomposite structure. It is known that the removal of the template from the polymer network creates smart cavities that allow us to know the shape and chemical functionality of the template Cu^2+^ [46]. In contrast, for the NIP/Mt adsorbent, there is no significantly distinct adsorption is noted for copper. As a matter of fact, one can note in Figure 5b that the maximum adsorption is noted for zinc and not copper. One can even note that the NIP adsorbs more competitive ions (Zn^2+^, Pb^2+^ and Fe^3+^) than the IIP due to non-specific adsorption. From Figure 5a,b, clearly it is essential to fabricate a copper ion imprinted polymer in order to achieve selective removal of this template ion; herein, IIP/Mt takes up about 1.7 times more copper than NIP/Mt. This ratio is not that large but the copper ion imprinting strategy results in a capture of copper ions three times higher than that of the other metal ions.

To study the adsorption process, pseudo-first-order and pseudo-second-order and Elovich models were used to control the check adsorption kinetics of IIP/Mt and NIP/Mt.

The pseudo-first-order and pseudo-second-order and Elovich models are described as Equations (5) [47], (6) [48] and (7) [49], respectively:

(5) $$log\left(q_{e}-q_{t}\right)=\log q_{e}-\frac{k_{1}}{2.303}t$$

(6) $$\frac{t}{q}=\frac{1}{k_{2}q_{e}^{2}}+\frac{t}{q_{e}}$$

(7) $$q_{t}=\frac{1}{b}\ln\left(ab\right)+\frac{1}{b}\ln t$$

where *q_t_* (mg/g) and *q_e_* (mg/g) are the adsorption capacities of metal ions on IIP/Mt and NIP/Mt at time *t* and equilibrium, respectively; *k*_1_ (L/min) and *k*_2_ (g/mg min) are the first-order and second-order adsorption constants, respectively; *a* represents the initial adsorption rate, whereas *b* is a constant related to the surface coverage. Actually, the Elovich model has been found to fit adsorption kinetics of MIP-coated silica beads [50], for this reason it was also considered in this work.

**Figure 6:**
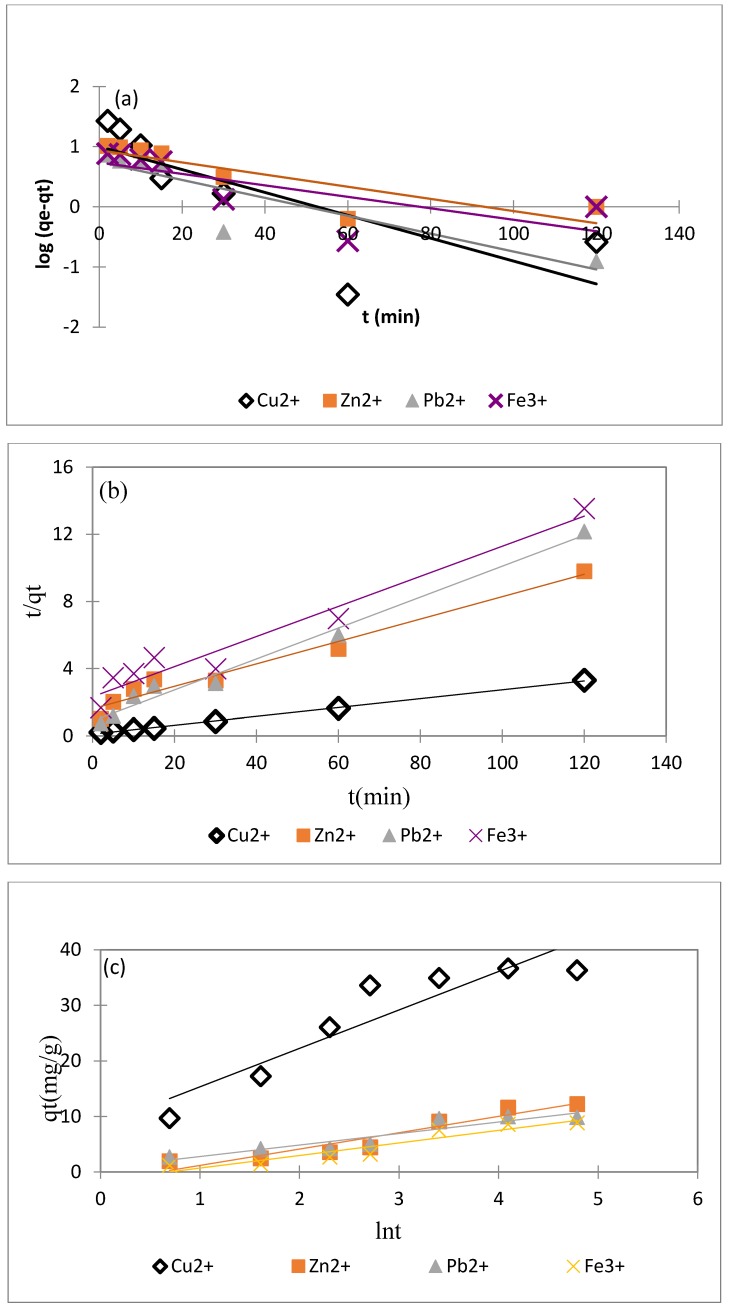
Pseudo-first-order (**a**), and pseudo-second-order (**b**) and Elovich (**c**) kinetics models applied to the adsorption of Cu^2+^, Zn^2+^, Pb^2+^ and Fe^3+^ onto IIP/Mt nanocomposites.

The kinetic modeling for the adsorption of metal ions on IIP/Mt nanocomposites is shown in Figure 6 and the adsorption kinetics constants and the correlation coefficient values *R*^2^ are summarized in Table 1. The values of the correlation coefficient *R*^2^ of the pseudo-second-order model are significantly higher than those of the other models and closer to unity and the computed equilibrium *q_e_* values are matching those determined experimentally. This thus accounts for pseudo-second-order mechanism of the adsorption process on the IIP/Mt nanocomposite. It follows that chemisorption is the driving force for the adsorption of copper on the IIP/Mt nanocomposite [51].

The kinetic results were also analyzed using the Webber and Morris intraparticle diffusion model [52]:

(8) $$q_{t}=K_{i}\;t^{0.5}+C,$$

where *K_i_* is the intraparticle diffusion rate constant (mg/(g min^0.5^)), *q_t_* is the amount of solute adsorbed per unit mass of adsorbent at a time *t* and *C* is the interception related to the thickness of the limit layer.

**Figure 7:**
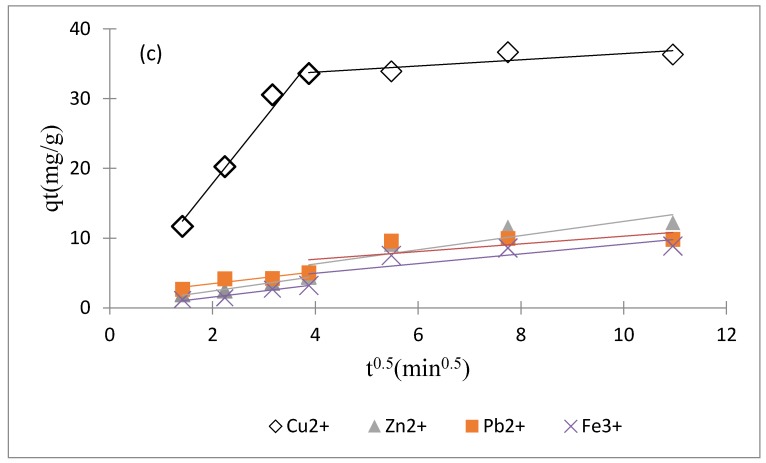
Intraparticle diffusion kinetics for adsorption of Cu^2+^, Zn^2+^, Pb^2+^, and Fe^3+^ onto IIP/Mt nanocomposites.

Figure 7 shows a graphical representation of *q_t_* versus of *t*^0.5^ for the metal ions Cu^2+^, Zn^2+^, Pb^2+^, and Fe^3+^, which yields two plots for each metal ion. The first linear portion represents the instantaneous adsorption step; the rapid adsorption rate of Cu^2+^ is due to the availability of free sites on the external surface. The second linear portion is due to intraparticle diffusion, which represents the limiting step of the adsorption process. The second linear portion is almost horizontal, probably due to saturation, and the longer time required to reaching equilibrium. This second portion of the plots in Figure 7 does not have a zero intercept so intraparticle diffusion is not the only determining step [53].

Table 2 presents the correlation coefficient values, the constant C, and the intraparticle diffusion constants (*K_i_*), which are calculated using Equation (8). The values reported in Table 2 clearly indicate that the diffusion rate constant (*K_i_*_1_) in the first step is higher than in the second step (*K_i_*_2_).

#### 3.3.3. Adsorption Isotherms

**Figure 8:**
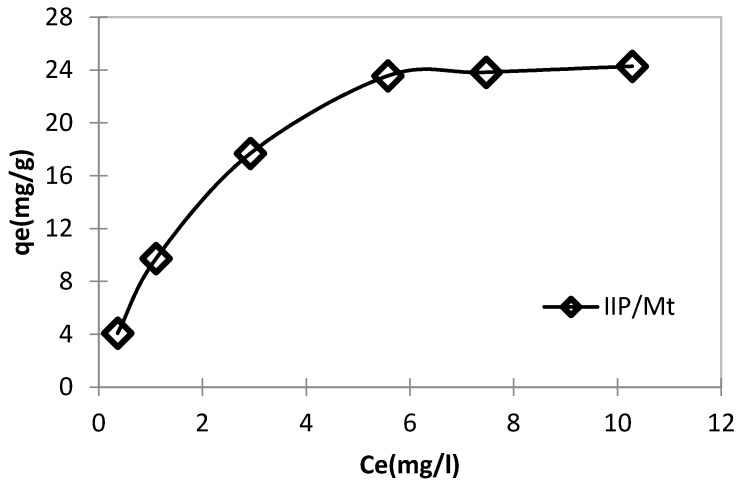
Adsorption isotherms of Cu^2+^ on IIP/Mt nanocomposite at pH 5.

**Figure 9:**
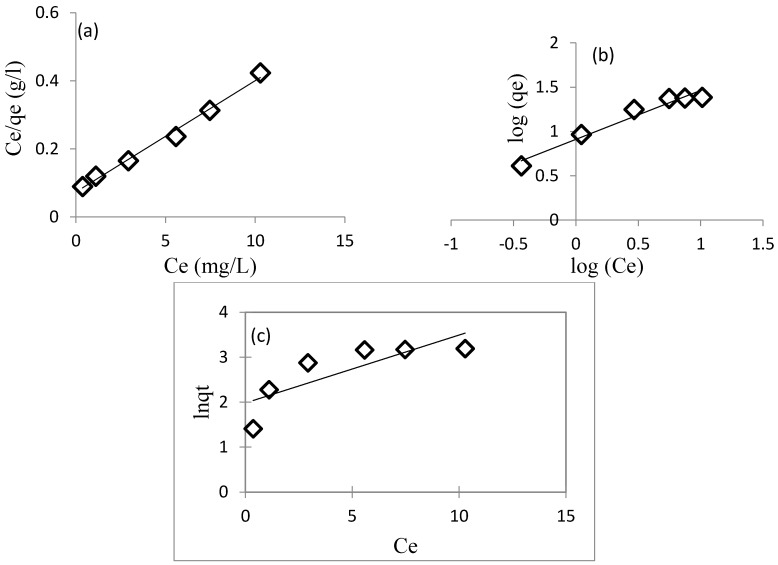
Isotherms models for adsorption of Cu^2+^ on IIP/Mt at room temperature: Langmuir (**a**), Freundlich (**b**), and Jovanovic (**c**).

Figure 8 shows the effects of aqueous phase Cu^2+^ concentrations on equilibrium adsorption capacity on IIP/Mt. The adsorption capacity increases with increasing concentration in the aqueous phase. Adsorption of the metal ion saturates at 23.562 mg/g (*q_max_*). In this study the adsorption isotherm is applied to study the interactions between metal ions and the active sites of our adsorbent (IIP/Mt). The adsorption isotherm is expressed for an adsorbent-adsorbate pair as a function of concentration. The adsorption isotherm of Cu^2+^ ions was checked whether it fits the Langmuir [54], Freundlich [55] or Jovanovic [56] isotherm models (see Figure 9) as described by Equations (9)–(11), respectively. The Langmuir isotherm theory assumes that adsorption is single-layer and takes place at homogeneous sites specific to the adsorbent and the Freundlich isotherm assumes that adsorption is multi-layer and that the surface of the adsorbent is heterogeneous. The Jovanovic model assumes mechanical contacts likely to occur between adsorbing and desorbing molecules [57].

(9) $$\frac{C_{e}}{q_{e}}=\frac{1}{QmK_{L}}+\frac{C_{e}}{Q_{m}}$$

(10) $$logq_{e}=logK_{F}+\frac{1}{n}logC_{e}$$

(11) $$lnq_{t}=lnq_{m}-K_{j}\;C_{e},$$

where *q_e_* (mg/g) is the adsorbed amount at equilibrium, *C_e_* is the equilibrium concentration of the metal ions (mg/L), *K_L_* (L/mg), *K_F_*$\left({\mathrm{mg}}^{1-1/n}/{\mathrm{gL}}^{1/n}\right)$, *K_j_* are the Langmuir equilibrium and the Freundlich and Jovanovic constants, respectively; *q_m_* the maximum adsorption capacity (mg/g), *n* is the heterogeneity factor, and 1/*n* value is related to the sorption intensity.

Table 3 lists all isotherm parameters. The data show that the Langmuir model (Figure 8a) is more suitable to describe the adsorption reaction of Cu^2+^ on the IIP/Mt with the experimental data with higher *R*^2^ values. Note that the Jovanovic constant is negative which has no physical meaning. Therefore, these results imply that the adsorption process of IIP/Mt is surface monolayer adsorption and the adsorption sites are homogeneous [58,59], which is in line with the plateau noted for the direct adsorption isotherm displayed in Figure 8.

#### 3.3.4. Adsorption Selectivity

To evaluate the selective properties of the IIP/Mt and NIP/Mt nanocomposites, adsorption of Cu^2+^ was conducted in the presence of competitive ions in binary systems. In our work, the binary solute solutions, including Cu^2+^/Zn^2+^, Cu^2+^/Pb^2+^ and Cu^2+^/Fe^3+^ were investigated to assess the selectivity to template Cu^2+^ in conditions where ions are simultaneously present in the aqueous solutions. The distribution coefficients (*K_d_*), the selectivity coefficients (*K*) and the relative selectivity coefficients (*K′*) were calculated (see Table 4). It is noted that the distribution coefficient value of Cu^2+^ is greater than that of the other metal ions. Therefore, high selectivity coefficients were determined for IIP/Mt and NIP/Mt nanocomposites: 10.308, 6.515 and 8.524 for Cu^2+^/Zn^2+^, Cu^2+^/Pb^2+^ and Cu^2+^/Fe^3+^, respectively. The selectivity for Cu^2+^ is due to the ion imprints shaped in the polymer network of the nanocomposite. The geometry, charge and size of the prints account for the selective recognition of Cu^2+^ ions over the other cationic competitors.

#### 3.3.5. Regeneration of IIP/Mt

**Figure 10:**
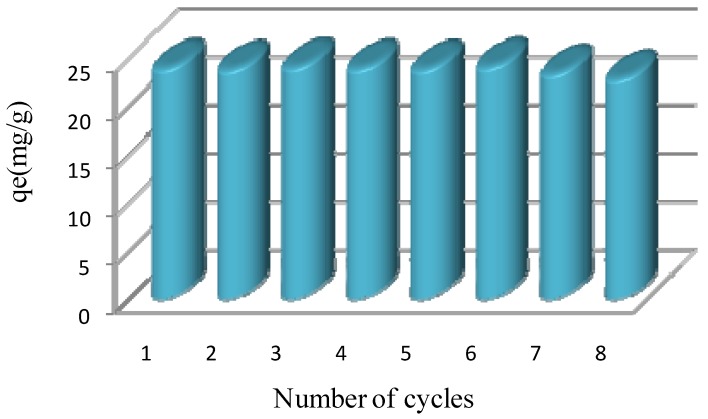
Recycling of the IIP/Mt in the removal of Cu^2+^ from aqueous relations (*C*_0_ = 20 mg/L, pH = 5, RT).

In order to evaluate the reusability of the synthesized sorbent, several adsorption-desorption cycles were carried out on the same sample of IIP/Mt nanocomposite at the same Cu^2+^ concentration of 20 mg/L. The desorption of Cu^2+^ ions adsorbed from the IIP/Mt nanocomposites was carried out using a 2 M HNO_3_ solution. The results displayed in Figure 10 indicate the stability of the IIP/Mt nanocomposite adsorbent, which could be regenerated without significant loss of performance. Indeed, up to eight adsorption/desorption cycles were applied to the robust adsorbent designed, which so far has not shown any signs of decrease in performance.

## 4. Conclusions

Montmorillonite-based ion imprinted polymer nanocomposite was prepared by radical photopolymerization under visible light exposure. The pre-polymerization complex was prepared by mixing Cu(NO_3_)_2_ and functional monomers of 4-VP and MAA mixed in DMF/H_2_O. Then, this solution was mixed with the crosslinker (EDGMA), photoinitiator (camphorquinone, CQ), and modified montmorillonite by diazonium (Mt_DZ). We show that diazonium salt is a good intercalant for clay and for triggering in situ radical photopolymerization. In this study, Cu^2+^-imprinted polymer/montmorillonite nanocomposite was successfully prepared and applied for the pre-concentration and determination of Cu^2+^ ions in aqueous solutions. The experimental data followed the Langmuir isothermal model and pseudo-second-order kinetics. In addition, these artificial sites shaped to accommodate Cu^2+^ could uptake copper at high extent compared to the competing metal ions Zn^2+^, Pb^2+^, and Fe^3+^, but also much more than non-imprinted polymer/montmorillonite nanocomposite. The selectivity with other ions confined that IIP/Mt showed high specific Cu^2+^ ions.

This work demonstrates for the first time the efficiency of diazonium salt to initiate visible light radical photopolymerization within the interlayer spacings of layered aluminosilicates. The method could be applied to other nanomaterials that could serve as nanoscale platforms to be coated by ion or molecularly imprinted polymers.

## Figures and Tables

**Table 1:** Kinetic parameters of the pseudo-first-order and the pseudo-second-order and Elovich rate equations for ions metals adsorption on IIP/Mt nanocomposites.

Ions	Pseudo-First-Order			Pseudo-Second-Order			Elovich		
	*k*_1_ (L/min)	*q_e_* (mg/g)	*R^2^*	*k*_2_·10^−2^ (g/mg min)	*q_e_* (mg/g)	*R^2^*	*a* (mg/g min)	*b* (g/mg)	*R^2^*
Cu^2+^	0.044	9.977	0.591	0.625	38.461	0.998	8.568	0.144	0.855
Zn^2+^	0.023	8.689	0.728	0.269	15.151	0.966	2.620	0.340	0.907
Pb^2+^	0.032	5.534	0.782	0.945	10.869	0.984	2.924	0.482	0.865
Fe^3+^	0.021	5.457	0.519	0.340	11.236	0.954	0.368	0.441	0.893

**Table 2:** Intraparticle diffusion constants and correlation coefficients for the adsorption of Cu^2+^, Zn^2+^, Pb^2+^, and Fe^3+^ ions on IIP/Mt nanocomposite.

Ions	Intraparticle Diffusion Model Constants					
	*K_i_*_1_	*C*_1_	(*R*_1_)^2^	*K_i_*_2_	*C*_2_	(*R*_2_)^2^
Cu^2+^	9.221	−0.597	0.977	0.442	32.031	0.730
Zn^2+^	1.037	0.349	0.980	1.017	2.221	0.782
Pb^2+^	0.850	1.777	0.862	0.551	4.764	0.501
Fe^3+^	0.885	−0.225	0.951	0.692	2.199	0.658

**Table 3:** Adsorption equilibrium constants for Langmuir and Freundlich and Jovanovic isotherms.

Adsorbents	Metal Ion	Langmuir Isotherm			Freundlich Isotherm			Jovanovic		
IIP/Mt	Cu^2+^	*q_m_* (mg/g)	*K_L_* (L/mg)	*R* ^2^	*K_F_* $\left({\mathrm{mg}}^{1-1/n}/{\mathrm{gL}}^{1/n}\right)$	*n*	*R* ^2^	*q_m_* (mg/g)	*K_j_*	*R* ^2^
		31.25	3.555	0.991	8.128	1.811	0.958	5.385	−0.151	0.666

**Table 4:** Selectivity adsorption parameters for IIP/Mt and NIP/Mt nanocomposites.

Metals	IIP/Mt		NIP/Mt		*K*′
	*k_d_*	*k*	*k_d_*	*k*	
**Cu^2+^**	29.904		2.073		
**Zn^2+^**	3.657	8.2	21.029	0.099	83.0
**Pb^2+^**	3.555	8.4	28.808	0.072	117
**Fe^3+^**	8.152	3.7	7.269	0.285	12.9
